# Decreased expression of the NF-κB family member RelB in lung fibroblasts from Smokers with and without COPD potentiates cigarette smoke-induced COX-2 expression

**DOI:** 10.1186/s12931-015-0214-6

**Published:** 2015-05-06

**Authors:** Jared A Sheridan, Michela Zago, Parameswaran Nair, Pei Z Li, Jean Bourbeau, Wan C Tan, Qutayba Hamid, David H Eidelman, Andrea L Benedetti, Carolyn J Baglole

**Affiliations:** Department of Medicine, 1001 Decarie Blvd, Montreal, QC H4A 3J1 Canada; Research Institute of the McGill University Health Centre, 1001 Decarie Blvd, Montreal, QC H4A 3J1 Canada; Department of Epidemiology and Biostatistics, 1001 Decarie Blvd, Montreal, QC H4A 3J1 Canada; Respiratory Epidemiology and Clinical Research Unit, Montreal Chest Institute, McGill University, Montreal, QC Canada; Department of Medicine, McMaster University, Hamilton, ON Canada; The UBC James Hogg Research Centre, University of British Columbia, Vancouver, BC Canada

**Keywords:** Inflammation, NF-κB, miRNA, COPD, Cigarette smoke, Lung

## Abstract

**Background:**

Heightened inflammation, including expression of COX-2, is associated with COPD pathogenesis. RelB is an NF-κB family member that attenuates COX-2 in response to cigarette smoke by a mechanism that may involve the miRNA miR-146a. There is no information on the expression of RelB in COPD or if RelB prevents COX-2 expression through miR-146a.

**Methods:**

RelB, *Cox-2* and miR-146a levels were evaluated in lung fibroblasts and blood samples derived from non-smokers (Normal) and smokers (At Risk) with and without COPD by qRT-PCR. RelB and COX-2 protein levels were evaluated by western blot. Human lung fibroblasts from Normal subjects and smokers with and without COPD, along with RelB knock-down (siRNA) in Normal cells, were exposed to cigarette smoke extract (CSE) *in vitro* and COX-2 mRNA/protein and miR-146a levels assessed.

**Results:**

Basal expression of RelB mRNA and protein were significantly lower in lung cells derived from smokers with and without COPD, the latter of which expressed more *Cox-2* mRNA and protein in response to CSE. Knock-down of RelB in Normal fibroblasts increased *Cox-2* mRNA and protein induction by CSE. Basal miR-146a levels were not different between the three groups, and only Normal fibroblasts increased miR-146a expression in response to smoke. There was a positive correlation between systemic RelB and *Cox-2* mRNA levels and circulating miR-146a levels were higher only in GOLD stage I subjects.

**Conclusions:**

Our data indicate that RelB attenuates COX-2 expression in lung structural cells, such that loss of pulmonary RelB may be an important determinant in the aberrant, heightened inflammation associated with COPD pathogenesis.

## Introduction

Chronic obstructive pulmonary disease (COPD) is a heterogeneous disease associated with an enhanced, chronic inflammatory response due to exposure to noxious particles or gases. Cigarette smoke is the single greatest risk factor for developing COPD, with an estimated 80-90% of COPD cases being due to chronic smoke exposure [1,2]. Cigarette smoke contributes to COPD by inciting inflammation, recruiting T cells, macrophages and neutrophils to the lung in part via the induction of inflammatory mediators, including cyclooxygenase-2 (COX-2) [3-5]. COX-2 catalyzes the transformation of arachidonic acid (AA) into thromboxanes and prostaglandins (PG) such as PGE_2_, an immunoregulatory PG that is elevated in COPD subjects [5,6]. Inhibition of COX-2-derived PGE_2_ also protects against the development of emphysema [7], supporting a role for COX-2 in the pathobiology of COPD. Numerous cell types within the lung are capable of producing COX-2 in response to smoke, including lymphocytes, epithelial cells, smooth muscle cells and fibroblasts [4,8,9]. Although the primary role of the fibroblast is to synthesize and maintain extracellular matrix (ECM), thereby providing structure and support to the lung, there is now ample evidence that fibroblasts contribute to chronic inflammation by producing an array of cytokines, chemokines, lipid mediators and proteases [10-12]. Fibroblasts are among the first cells in the lung which encounter cigarette smoke, potently activating them to increase the production of IL-8, COX-2 and other mediators involved in leukocyte recruitment [4,13,14]. Alveolar fibroblasts also provide connection between type II pneumocytes and endothelial cells, thereby providing migratory leukocytes with a directional conduit [15]. Thus, disordered fibroblast behaviour may regulate the switch to chronic, persistent inflammation in part by their ability to promote the recruitment, survival and retention of leukocytes and other immune cells to target organs such as the lung due to chronic smoke exposure [10,16,17].

Lung fibroblasts derived from patients with COPD have high intercellular adhesion molecule-1 (ICAM-1) expression and secrete more IL-6, IL-8, MMP-9 and PGE_2_ [2,16,18], the latter being due to increased COX-2 protein expression [19]. The induction of COX-2 by cigarette smoke is regulated at least partially by the transcription factor nuclear factor-κB (NF-κB) [4]. Activation of the canonical NF-κβ pathway- typically composed of RelA and p50 subunits- increases gene transcription and the expression of inflammatory proteins including IL-6, IL-8 and COX-2. However, activation of this NF-κB pathway also increases RelB expression [20], the key protein in the alternative NF-κB pathway. RelB is sequestered in the cytosol by the inhibitory NF-κB protein p100. Signal-specific processing of p100 by the NF-κB-inducing kinase (NIK) produces p52, which together with RelB translocate to the nucleus to regulate gene expression [21]. Regulation of the alternative NF-κB pathway during inflammatory conditions (TNF-α, LPS, cigarette smoke, *etc.*) can occur via stabilization of NIK, which increases NIK expression to allow efficient processing of p100 to p52 [22]. Inflammatory stimuli can also lead to the upregulation of RelB expression, enhanced nuclear localization [20,23,24] or processing/cleavage of RelB protein [25,26]. Many of these may contribute to the ability of RelB to modulate chronic inflammation. In this regard, RelB prevents persistent non-infectious inflammation in the liver and lung, a phenomena attributed to the suppressive abilities of RelB in non-lymphoid tissue, possibly fibroblasts [27,28]. We have shown that RelB dampens cigarette smoke-induced pulmonary inflammation both *in vitro* and *in vivo*, including the expression of COX-2 [29,30]. COX-2 is over-expressed by COPD lung fibroblasts [19] and we have shown that RelB protein is degraded by cigarette smoke [29], rendering it possible that heightened smoke-induced COX-2 expression in COPD is associated with absent/low RelB expression. Moreover, whether systemic RelB expression is also altered as a consequence of chronic smoke exposure or COPD severity (*i.e.* GOLD stage) or is associated with the expression of *Cox-2* is also not known.

We recently published that in murine lung fibroblasts, RelB suppression of cigarette smoke-induced COX-2 expression is due to up-regulation of the microRNA-146a (miR-146a) [31]. miRNAs are single-stranded, non-coding, 22 nucleotide-long RNA which act posttranscriptionally to inhibit protein expression [32] and are of increasing interest as biomarkers for COPD [33]. Cigarette smoke alters lung structural cell and circulating miRNA levels, including miR-146a [31,34,35], an anti-inflammatory miRNA that under-expressed in cytokine-stimulated lung fibroblasts derived from COPD patients, which ultimately results in higher COX-2 expression [36]. We postulate that altered RelB expression in COPD-derived lung fibroblasts facilitates cigarette smoke-induced COX-2 due to dysregulation of miR-146a expression. Given that there is high correlation of expression between immune cells and circulating miRNA expression levels [37] and systemic expression miR-146a are now indicted in several diseases including rheumatoid arthritis [37], we also postulated that miR-146a levels would be associated with clinical features of COPD in relation to changes in circulating RelB expression.

Therefore, we first sought to investigate whether down-regulation of RelB expression by cigarette smoke renders lung cells unable to increase miR-146a levels, thereby potentiating the induction of COX-2. Herein we show for the first time that fibroblasts derived from smokers with and without COPD have significantly lower RelB mRNA and protein expression compared to Normal (non-smoker) fibroblasts. RelB expression decreases in Normal fibroblasts exposed to cigarette smoke *in vitro*, and siRNA knock-down of RelB potentiates *Cox-2* mRNA and protein induction by cigarette smoke in Normal fibroblasts. Utilizing blood samples from subjects participating in the Canadian Chronic Obstructive Lung Disease (CanCOLD) platform [38], we found that there was a significant positive correlation between systemic RelB and *Cox-2* mRNA expression. Collectively, our results highlight the differential regulation of RelB expression between lung structural cells and peripheral blood and suggest that dysregulation of RelB levels within pulmonary structural cells may be a contributing factor in the heightened inflammatory response that is characteristic of individuals who smoke.

## Materials and methods

### Materials

All chemicals were purchased from Sigma (St. Louis, MO) except MG-132 which was from Tocris Bioscience (Minneapolis, MN).

### Cell culture

**Figure 1:**
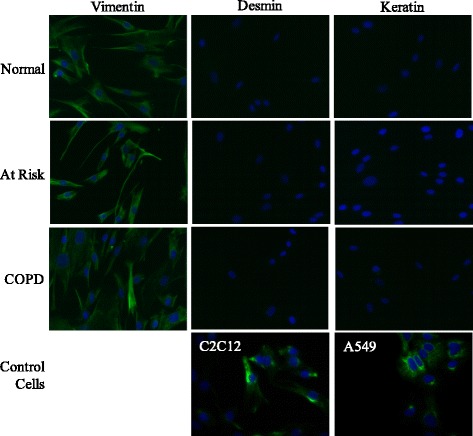
Characterization of human lung fibroblasts. Representative immunofluorescence images of human lung fibroblasts derived from Normal (non-smoker), At Risk (Smoker without COPD) and COPD subjects. Target proteins were visualized with fluorescein (green) in combination with Hoechst (blue) nuclear stain; only the merge images are shown. C2C12 (myoblasts) and A549 (epithelial) cells were used as positive controls for desmin and keratin. Fibroblasts from each patient type were incubated with antibodies specific to vimentin (left), desmin (middle), and keratin (right). All human lung fibroblast derived by this method were positive for vimentin but negative for desmin and keratin and exhibited typical fibroblast morphology. Magnification = 20x. Representative images are shown.

Lung tissue was obtained from individuals undergoing lung resection surgery at McMaster University. Recruited individuals included those with COPD, subjects without COPD but who were current or former smokers (At Risk) or non-smokers without COPD (Normal). The clinical features of the subjects from which the lung fibroblasts were derived are given in Table 1. This study was approved by the Research Ethics Board of St Joseph’s Healthcare Hamilton and all patients gave written, informed consent. Primary lung fibroblasts were cultured as previously described [39] and only tissue from cancer-free regions was used for the derivation of fibroblasts. Prior to experimentation, fibroblasts were characterized based on morphology and vimentin expression as well as the absence of cytokeratin (epithelial cell marker), desmin (muscle cell marker) and α-smooth muscle actin (α-SMA; myofibroblast marker) [39] (Figure 1). Following characterization, cells were expanded and either frozen in liquid nitrogen or maintained in culture as a monolayer. For experimentation, primary fibroblasts were cultured in MEM (Life Technologies, Gaithersburg, MD) supplemented with 10% fetal bovine serum (FBS; HyClone Laboratories, Logan, UT) and incubated in humidified 5% CO_2_/95% air at 37°C. All fibroblast strains were used at the earliest possible passage. For assessment of basal expression levels, all available fibroblast strains were cultured and analyzed at the same time and were within 1 passage (passage 3–4). Additional experiments were conducted with fibroblasts from a minimum of three different individuals of each patient category.

**Table 1:** **Patient characteristics of fibroblast strains derived from lung resection**

	**Normal**	**At Risk**	**COPD**
**n of subjects**	6	15	12
**Age (yr)**	66.7 ± 2.6	64 ± 0.7	68.3 ± 0.9
**Gender (M/F)**	(2/4)	(8/7)	(8/4)
**Smoking** ^**#**^	0.0	36.5 ± 1.2	37.6 ± 1.3
**FEV**_**1**_ **(%)**	88.2 ± 3.3	88.2 ± 0.9	65.8 ± 2.16
**FVC (%)**	83 ± 3.4	92 ± 0.9	81.4 ± 2.3
**FEV**_**1**_ **/FVC (%)**	88 ± 2.7	76.6 ± 0.5	55.0 ± 1.7***

Results presented as average ± SEM.

^#^Denotes pack-years.

***FEV_1_/FVC of COPD patients was significantly lower (p < 0.05) compared to either the non-smokers or the smokers without COPD, which did not differ from each other.

### Canadian chronic obstructive lung disease (CanCOLD)

CanCOLD is a prospective longitudinal cohort study tracking 1800 subjects and comprises 2 COPD subsets (GOLD ≥2 and GOLD 1) and 2 subsets of non-COPD peers, *i.e*., normal post bronchodilator spirometry (ever smoker for those at-risk and never-smoker for the healthy controls), matched for sex and age. The CanCOLD cohort is described in detail in [38]. The CanCOLD study was approved by the REB at the McGill University Health Centre (MUHC) - Study # 09-025-BMC. Peripheral blood was collected using PAXgene blood RNA tubes (PreAnalytiX GmbH, Hombrechtikon, Germany) at initial visit and frozen at −80°C until analysis. Clinical characteristics of the subjects utilized in this part of the study are in Table 2.

**Table 2:** **Clinical characteristics of the CanCOLD Subjects**

**Variable**	**Normal**	**At Risk**	**GOLD 1**	**GOLD 2+**	**Overall P-values**
Age, in year [mean(sd)]	67.75 (5.84)	66.06 (8.44)	66.94 (11.20)	67.67 (10.22)	0.952
Sex, male gender [n(%)]	4 (25.00)	5 (31.25)	9 (56.25)	9 (50.00)	0.215
Tobacco smoking status [n(%)]					
Never	16 (100.00)	-	7 (43.75)	4 (22.22)	<0.001*
Ex-smokers	-	14 (87.50)	6 (37.50)	9 (50.00)	<0.001*
Current smokers	-	2 (12.50)	3 (18.75)	5 (27.78)	0.149
Cigarette smoker pack-years [mean(sd)]	-	14.56 (11.26)	20.02 (26.57)	27.28 (28.78)	<0.001*
Post-bronchodilator spirometry [mean(sd)]					
FEV_1,_ L	2.38 (0.48)	2.55 (0.63)	2.59 (0.56)	1.60 (0.70)	<0.001*
FEV_1_, % predicted	101.28 (11.33)	100.04 (10.75)	101.06 (20.55)	60.62 (16.68)	<0.001*
FEV_1_/FVC, %	76.39 (4.21)	78.23 (4.48)	67.04 (7.14)	53.18 (12.93)	<0.001*
Biomarker expression [mean(sd)]					
RelB (X10^−3^)	0.74 (1.08)	0.61 (0.19)	0.61 (0.28)	0.56 (0.16)	0.326
COX-2 (X10^−3^)	7.74 (8.35)	5.62 (1.94)	6.30 (2.74)	6.76 (2.95)	0.453
146a (X10-3)	0.09 (0.11)	0.46 (1.10)	0.47 (0.62)	1.22 (3.37)	0.064

*indicates significance between the groups for the clinical characteristics in each row.

### Preparation of cigarette smoke extract (CSE)

Research grade cigarettes (3R4F) with a filter were obtained from the Kentucky Tobacco Research Council (Lexington, KT) and CSE generated as previously described [29,40-42]. Briefly, CSE was prepared by bubbling smoke from two cigarettes into 20 ml of serum-free MEM, the pH adjusted to 7.4, sterile- filtered with a 0.45-μm filter (25-mm Acrodisc; Pall Corp., Ann Arbor, MI) and was used within 30 minutes of preparation. An optical density of 0.65 (320 nm) was considered to represent 100% CSE and was diluted in serum-free MEM to 2% CSE, a concentration which potently increases COX-2 expression in primary lung fibroblasts deficient in RelB expression [31]. For comparison of the cigarette smoke response between the three patient groups, experiments were conducted with fibroblasts cultured at the same time utilizing the same CSE preparation to minimize experimental variability.

### Analysis of gene expression

Total RNA was isolated from media- or CSE-treated fibroblasts using a Qiagen miRNeasy kit (Qiagen Inc., Valencia, CA). For processing the CanCOLD samples, total RNA was isolated using PAXgene blood RNA kit (PreAnalytiX GmbH) according to the supplier’s protocol. In each case, RNA was eluted in 30 μl RNase-free water and RNA content and purity was measured using a Nanodrop 1000 spectrophotometer (Thermo Fisher Scientific, Wilmington, DE). Reverse transcription of total RNA was carried out in a 20-μl reaction mixture by iScript II^TM^ Reverse Transcription Supermix (Bio-Rad Laboratories, Mississauga, Ontario) at 25°C for 5 min, at 42°C for 30 min and at 85°C for 5 min. Real time (qPCR) was performed with 1 μl cDNA and 0.5 μM primers added in Ssofast^TM^ Eva Green® Supermix (Bio-Rad) and PCR amplification was performed using a CFX96 Real-Time PCR Detection System (Bio-Rad). Primer sequences for human *Cox-2* are TCACAGGCTTCCATTGACCAG (f) and CCGAGGCTTTTCTACCAGA (r) and for human RelB are TGTGGTGAGGATCTGCTTCCAG (f) and GGCCCGCTTTCCTTGTTAATTC (r). Thermal cycling was initiated at 95°C for 30 sec and followed by 40 cycles of denaturation at 95°C for 30 s and annealing for 5 s. Melt curves were obtained to ensure that nonspecific products were absent. The fluorescence detection threshold was set above the non-template control background within the linear phases of PCR amplifications and the cycle threshold (Ct) of each reaction was detected. Gene expression data were analyzed using the ΔΔCt method normalized to housekeeping (β-actin).

### Analysis of miR-146a expression

miRNA expression was assessed by two-step TaqMan® RT-PCR (Applied Biosystems, Carlsbad, CA) for miR-146a and U6 snRNA, a small nuclear RNA (snRNA) used as an internal control for miRNA analysis [43,44]. miRNA expression was normalized to the U6 snRNA levels and fold-change was determined using 2^−ΔΔCt^ method as we have described [31,45].

### Western blot

Fibroblasts were grown to approximately 90% confluence before being treated with CSE. In separate experiments, Normal fibroblasts were pretreated with the proteasome inhibitor MG-132 (10 μM) for 2 hours prior to addition of 2% CSE. Total cellular protein was prepared using 1% IGEPAL lysis buffer [40] and 5–10 μg of protein were fractionated on SDS-PAGE gels and electro-blotted onto Immun-blot PVDF membrane (Bio-Rad Laboratories, Hercules, CA). Antibodies against RelB (1:1000; Cell Signaling), COX-2 (1:1000) (Cayman Chemical, Ann Arbor, MI), p65 (1:1000, Santa Cruz) and total Actin (1:50,000; Milipore, Temecula, CA) were used to assess changes in relative expression. Proteins were visualized using HRP-conjugated secondary antibodies (1:10,000) followed by enhanced chemiluminescence (ECL) and imaged using a ChemiDoc™ XRS+ System (Bio-Rad).

### RelB knock-down in primary lung fibroblasts

Normal fibroblasts (non-smoker) were seeded at 1–2 x 10^4^ cells/cm^2^ and transfected with 40 nM of siRNA against RelB (Santa Cruz, Catalogue number sc-36402) or non-targeting control siRNA (Santa Cruz, Catalogue number sc-37007) according to manufacturer’s instructions. Six hours after the transfection, the cells were switched to serum-free MEM. On the next day, cells were treated with 2% CSE for 3–24 hours and RNA or protein collected for further analysis as described above. Verification of RelB knock-down was done by western blot.

### Immunocytochemistry

Lung fibroblasts from Normal, At Risk and COPD subjects were cultured on glass chamber slides and left untreated or were treated with 2% CSE. Following treatments, cells were washed once with PBS/Tween, permeabilized/fixed using 3% H2O2/methanol for 10 min, and blocked with Universal Blocking Solution for 1 hour at room temperature. The antibodies against RelB (1:300) and p65 (1:200) were diluted in Antibody Diluent Solution (Dako) and incubated overnight at 4°C. Alexa Fluor-555 anti-goat or anti-rabbit IgG antibody was used for secondary binding (1:1000) and incubated for 1 hour at room temperature. Slides were then mounted in ProLong® Gold Anti-Fade (Invitrogen), viewed on an Olympus IX71 fluorescent microscope (Olympus, Ontario, Canada) and photographed using a Retiga 2000R camera with ImagePro Plus software. Fluorescent images of nuclei are visualized by Hoechst staining (1:2000). All procedures were performed at the same time to minimize variability in fluorescence intensity.

### Statistical analysis

For experimental data utilizing human lung fibroblasts, statistical analysis was performed using GraphPad Prism 6 (v. 6.02; La Jolla, CA). A two-way analysis of variance (ANOVA) followed by a Newmann-Keuls test was used to assess differences between treatment groups of more than two factors when grouped by two variables unless otherwise indicated. A one-way analysis of variance (ANOVA) followed by a Newman-Keuls multiple comparisons test was used to assess differences in baseline values between the three subject groups. Statistical analysis of blood mRNA/miRNA levels with clinical parameters was analyzed on SAS version 9.3 (SAS Institute. Inc., Cary, N.C). Descriptive data were summarized using means and STD distributions or counts and percentages for the four study groups. Statistically significant differences among the four groups were then compared by using ANOVA analysis (or their non-parametric equivalence-Kruskal-Wallis Test) and the Chi-square test as appropriate. Analysis of the correlation for each of the biomarker expression (*Cox-2*, RelB, miR-146a) with other biomarkers as well as clinical variables was performed using Pearson’s correlation coefficient. Results are expressed as the mean ± SEM or SD. In all cases, a p value < 0.05 is considered statistically significant.

## Results

### Lung fibroblast characterization

The clinical features of the subjects from which the lung fibroblasts were derived are given in Table 1. The FEV_1_/FVC ratio after bronchodilators was significantly lower in the COPD patients compared to either Normal (non-smokers) or smokers without COPD (At Risk). Non-smokers were identified as those individuals who were never-smokers (0 pack-years). There was no significant difference in pack-years between the smokers with and without COPD. All primary lung fibroblast used in this study had typical fibroblast morphology (flat, elongate with oval nuclei) and expressed vimentin (Figure 1). No staining was observed for cytokeratin or desmin indicating that the cultured fibroblasts did not contain cells of epithelial or muscle origin.

### RelB mRNA and protein expression is decreased in At Risk- and COPD-derived lung fibroblasts

**Figure 2:**
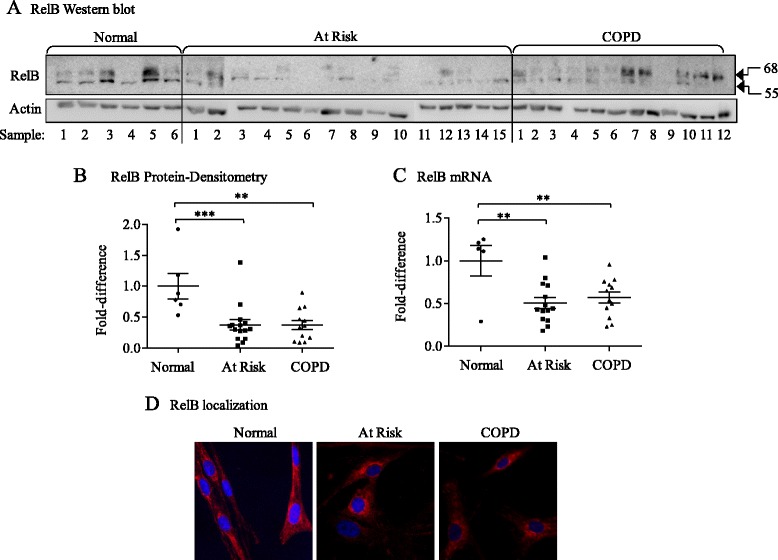
Reduced RelB mRNA and protein expression in human lung fibroblasts from At Risk and COPD subjects. **(A)** RelB western blot- RelB protein expression was detected in most lung fibroblasts examined at the predicted MW of 68 kDa. A faster migrating band of ≈ 55 kDa was also detected. There was an apparent decrease in RelB protein levels in the majority of lung fibroblasts from At Risk and COPD subjects. Sample numbers refer to lung fibroblasts from different individuals (Normal = 6; At Risk = 15; COPD = 12). **(B)** RelB protein densitometry- there was a significant decrease in RelB protein expression in lung fibroblasts derived from the lungs of At Risk (0.41 ± 0.08) as well as COPD subjects (0.38 ± 0.07) (*p = 0.0022 compared to fibroblasts from Normal subjects). There was no significant difference in RelB levels between At Risk and COPD fibroblasts. Results are expressed as the mean ± SEM and each symbol represents fibroblasts from a different individual. Densitometry is based on the band detected at the predicted MW of 68 kDa. **(C)** RelB mRNA- Relative RelB mRNA expression was significantly lower in At Risk (0.5 ± 0.06) and COPD (0.57 ± 0.07) compared to Normal (1.0 ± 0.18; * p < 0.0044) fibroblasts. Results are expressed as the mean ± SEM (fold-change) of RelB levels normalized to the Normal fibroblasts. **(D)** RelB localization: Immunofluorescent imaging of quiescent lung fibroblasts revealed that the majority of RelB in Normal, at Risk and COPD lung fibroblasts was localized to the cytoplasm (red colour) with minimal RelB evident in the nucleus (blue colour). Note that there appeared to be qualitatively less RelB (based on intensity) in the At Risk and COPD-derived lung fibroblasts. Representative images based on two independent experiments.

Our published data show that RelB suppresses cigarette smoke-induced COX-2 protein expression [30]. RelB is degraded by cigarette smoke *in vitro* and *in vivo* [29,46], raising the possibility that reduced RelB expression due to cigarette smoke exposure contributes to heightened COX-2 expression in COPD. RelB protein at the predicted molecular weight (MW ≈ 68 kDa) [47,48] was detectable in most of the lung fibroblasts examined; also evident was a band with a lower MW of ≈ 55 kDa (Figure 2A, *arrows*) that are consistent in size with degradation products of RelB [49]. In fibroblasts derived from both At Risk and COPD subjects, there appeared to be lower levels of RelB. Densitometric analysis of RelB protein at the predicted size (MW ≈ 68 kDa, analyzed in all subsequent Figures) indicated that there was a significant decrease in RelB protein expression in human lung fibroblasts from At Risk and COPD subjects compared to Normal (Figure 2B). There was no significant difference in RelB protein between smokers with and without COPD. The relative expression level of RelB mRNA was also significantly different between Normal and either At Risk- and COPD-derived fibroblasts (Figure 2C). RelB was predominantly localized to the cytoplasm in Normal, At Risk and COPD-derived lung fibroblasts cultured under basal condition, which appeared noticeably reduced in the At Risk and COPD cells (Figure 2D). Collectively, these data indicate that cigarette smoke exposure is associated with reduced RelB expression.

### Regulation of RelB expression in human lung fibroblasts by CSE

**Figure 3:**
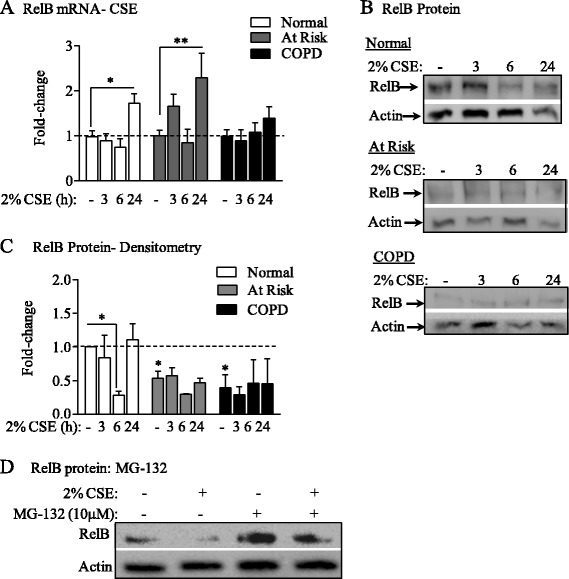
RelB protein expression is decreased by cigarette smoke exposure in Normal lung fibroblasts. Lung fibroblasts from Normal, At Risk and COPD subjects were exposed to 2% CSE for 3, 6, or 24 hours and RelB mRNA and protein levels evaluated. **(A)** RelB mRNA-CSE: RelB mRNA was increased in Normal and At Risk fibroblasts exposed to 2% CSE for 24 hours (*p < 0.05 and **p < 0.001). RelB mRNA levels were not significantly altered by CSE in COPD lung fibroblasts. Results are expressed as the mean ± SEM (fold-change) utilizing fibroblasts derived from 4–6 individual subjects for each group. **(B)** RelB protein: There was a noticeable decrease in RelB protein in Normal fibroblasts exposed to 2% CSE for 6 hours. Basal RelB protein levels were noticeably less in At Risk and COPD lung fibroblasts compared to Normal fibroblasts. Representative western blots are shown of three experiments. **(C)** RelB protein- Densitometry: Densitometric analysis of RelB expression revealed a significant difference in basal RelB protein expression between Normal and At Risk and COPD lung fibroblasts in the absence of 2% CSE (media only). RelB protein expression decreased in Normal lung fibroblasts at 6 hours of exposure to 2% CSE compared to media control (* p < 0.05). Results are expressed as the mean ± SEM (fold-change) utilizing fibroblasts derived from three individual subjects for each group. **(D)** RelB protein: MG-132: Normal lung fibroblasts were pretreated with MG-132 for 2 hours prior to cotreatment with 2% CSE for 6 hours and protein harvested for RelB expression. RelB levels were decreased following exposure to 2% CSE for 6 hours. MG-132 increased basal levels of RelB and prevented the loss of RelB after exposure to 2% CSE. Western blot image is representative of 3 separate experiments.

Exposure to cigarette smoke, but not CD40L, results in loss of RelB protein in murine lung fibroblasts [29]. RelB is degraded in a signal-specific manner in T cells [50], rendering it possible that chronic exposure to smoke contributes to the loss in RelB in At Risk and COPD lung fibroblasts. To evaluate this, we exposed lung fibroblasts to CSE via our *in vitro* model of smoke exposure [29,40,51]. In lung fibroblasts from both Normal and At Risk subjects, there was a significant increase in RelB mRNA following 24 hours of exposure to 2% CSE *in vitro* (Figure 3A, *white* and *grey bars*, respectively). In COPD lung fibroblasts, RelB mRNA levels remained unchanged throughout the 24-hour exposure time (Figure 3A, *black bars*). Consistent with the data presented in Figure 2, basal RelB protein expression remained significantly lower in At Risk and COPD fibroblasts cultured in a monolayer for the duration of the *in vitro* experiments (Figure 3B and C). RelB protein decreased in CSE-exposed Normal fibroblasts at 6 hours compared to media-only exposed cells (Figure 3B and C). There was no further change in RelB protein with 2% CSE exposure in fibroblasts from At Risk or COPD subjects. The reduction in RelB protein levels in Normal lung fibroblasts exposed to CSE was due to degradation by the proteasome. Treatment of fibroblasts with the cell-permeable and reversible proteasome inhibitor MG-132 [52,53] increased basal RelB protein and also attenuated the reduction in RelB protein upon exposure to 2% CSE (Figure 3D). These data support that exposure to cigarette smoke reduces the expression of RelB in lung fibroblasts via the proteasome degradation pathway.

### RelB attenuates cigarette smoke-induced COX-2 expression in primary lung fibroblasts

**Figure 4:**
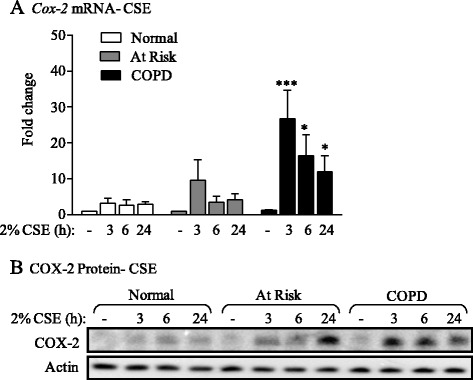
*Cox-2* mRNA and protein expression is induced by 2% CSE in Smoker and COPD lung fibroblasts. **(A)** *Cox-2* mRNA- CSE: There was a significant increase in *Cox-2* mRNA in COPD lung fibroblasts (*black bars*) exposed to 2% CSE for 3 hours (*** p <0.05 compared to respective control); there was a significantly more *Cox-2* mRNA in COPD fibroblasts at 6 and 24 hours of exposure. There was a trend towards increased *Cox-2* mRNA in At Risk fibroblasts (*grey bars*). Note the lack of relative *Cox-2* mRNA induction in Normal lung fibroblasts (*white bars*). Results are expressed as the mean ± SEM of 4–9 fibroblasts from each subject group. **(B)** COX-2 protein- CSE: There was a modest induction in COX-2 protein in Normal fibroblasts. There was noticeably more COX-2 protein in the At Risk and COPD fibroblasts. The induction in COX-2 protein occurred earlier COPD fibroblasts (by 3 hours). Representative western blot is shown of at least 3 different experiments.

Our published data utilizing RelB-deficient murine fibroblasts show that RelB potently suppresses cigarette smoke-induced COX-2 expression [31], leading us to speculate that lower RelB expression contribute to increased COX-2 in response to CSE. In response to 2% CSE, there was a transient increase in *Cox-2* mRNA in At Risk and COPD lung fibroblasts, with induction peaking at 3 hours of exposure (Figure 4A). The induction in *Cox-2* mRNA was significant in the COPD lung fibroblasts at all time-points examined compared to media-only (Figure 4A, *black bars*). There was minimal induction in *Cox-2* mRNA in Normal fibroblasts in response to CSE (Figure 4A, *white bars*) while *Cox-2* mRNA induction in Smoker fibroblasts was moderately higher (Figure 4B, *grey bars*). Exposure to 2% CSE also increased COX-2 protein expression only modestly in Normal fibroblasts but with noticeably higher induction occurring in the At Risk and COPD fibroblasts (Figure 4B), both of which also had significantly lower RelB protein levels (Figure 2).

**Figure 5:**
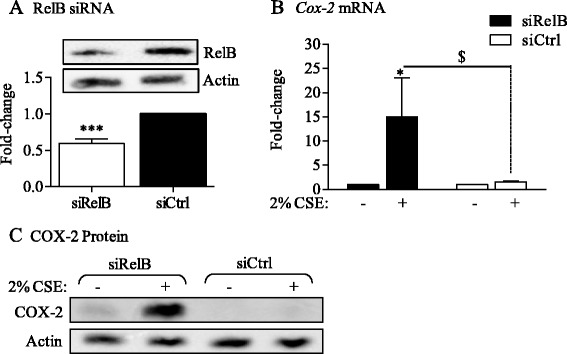
siRNA-mediated knock-down of RelB potentiates CSE-induced *Cox-2* mRNA and protein expression in Normal lung fibroblasts. **(A)** RelB siRNA: Lung fibroblasts derived from Normal subjects were transfected with siRNA against RelB (siRelB). RelB protein levels were decreased by approximately 40% compared to Ctrl-transfected (siCtrl; relative-change: 0.59 ± 0.065). Results are expressed as the mean ± SEM, n = 4 independent experiments*.* **(B)** *Cox-2* mRNA: Normal fibroblasts transfected with RelB siRNA were exposed to 2% CSE for 6 hours and *Cox-2* mRNA expression evaluated by qRT-PCR. There was a significant increase in *Cox-2* mRNA expression only when RelB expression is reduced (siRelB; fold-induction 15 ± 8; *p < 0.05 compared to RelB siRNA media only; $ p < 0.05 compared to Ctrl siRNA exposed to 2% CSE). Results are expressed as the mean ± SEM, n = 4–5 experiments*.* **(C)** COX-2 protein- there was a corresponding and dramatic increase in COX-2 protein in the siRelB fibroblasts exposed to 2% CSE compared to the siCTRL cells. Representative western blot is shown of at least two independent experiments.

To now test whether RelB is a factor that suppresses COX-2 induction upon direct smoke exposure, we used siRNA to knock-down RelB expression in Normal fibroblasts which express relatively high levels of RelB (Figure 2A). Following confirmation of successful reduction in RelB protein (Figure 5A), we first performed qPCR for *Cox-2* mRNA after exposure to 2% CSE. In Normal lung fibroblasts receiving Control siRNA (*i.e.* with RelB expression; siCtrl) there was low induction of *Cox-2* mRNA (Figure 5B, *open bars*). Attenuation of RelB expression via siRNA knockdown significantly increased *Cox-2* mRNA expression when cells were exposed to 2% CSE (Figure 5B, *black bars*). There was also a dramatic increase in COX-2 protein in RelB knockdown cells in response to 2% CSE (Figure 5C) supporting that the attenuation of cigarette smoke-induction of COX-2 *in vitro* in Normal lung fibroblasts is associated with RelB expression.

### p65 is similar between Normal, At Risk and COPD-derived lung fibroblasts

**Figure 6:**
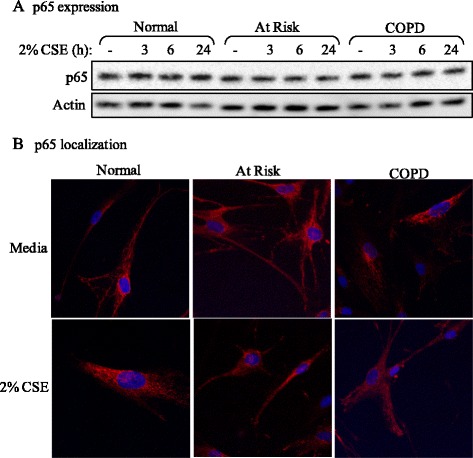
p65 expression and localization in Normal, At Risk and COPD lung fibroblasts. **(A)** p65 expression: There was no perceptible difference in p65 expression between Normal, At Risk and COPD-derived lung fibroblasts exposed to 2% CSE for up to 24 hours. Representative western blot is shown of 3 independent experiments. **(B)** p65 localization: There was little perceptible difference in the localization of p65 in lung fibroblasts from the three subject groups, which appeared predominantly cytoplasmic (red colour). Exposure to 2% CSE for 30 minutes did not appreciably alter the localization of p65. Representative images shown are based on two independent experiments.

The promoter of *Cox-2* contains two NF-κB binding sites and thus plays an important role in the transcriptional regulation of *Cox-2* expression via the classic (p65/p50) pathway [54]. It is possible therefore that the difference in *Cox-2* expression with CSE between At Risk and COPD (but with no difference in RelB protein- Figure 2) could be due to altered p65 expression or nuclear localization. Total p65 protein expression in lung fibroblasts from the three subject groups after exposure to 2% CSE for up to 24 hours was not noticeably different (Figure 6A). The localization of p65 in media only cells was largely cytoplasmic in fibroblasts from Normal, At. Risk and COPD subjects (Figure 6B). Exposure to 2% CSE for 30 minutes [29] marginally increased nuclear p65 in all three groups with little difference between them (Figure 6B). Therefore alterations in p65 expression or localization cannot account for the differential regulation of *Cox-2* between At Risk and COPD lung fibroblasts.

### Cigarette smoke induction of miR-146a in Normal human lung fibroblasts is independent of RelB expression

**Figure 7:**
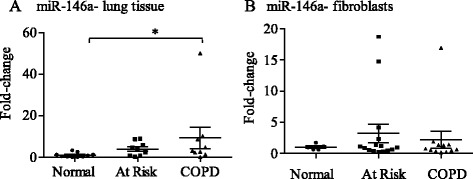
Basal miR-146a expression in human lung fibroblasts from Normal subjects as well as smokers with and without COPD. **(A)** miR-146a-lung tissue: There was a significant increase in miR-146a in lung tissue derived from COPD subjects compared to Normal. Results are expressed as mean ± SEM and each symbol represents a different individual (Normal, n = 10; At Risk, n = 9; COPD = 9). **(B)** miR-146a-fibroblasts: There was no significant difference in basal miR-146a expression in lung fibroblasts derived from the three patient groups. Results are expressed as the mean ± SEM of miR-146a levels normalized to the snRNA U6 and are expressed as fold-change compared to Normal fibroblasts. Each symbol represents fibroblasts from a different individual (Normal, n = 5; At Risk, n = 13; COPD = 11).

**Figure 8:**
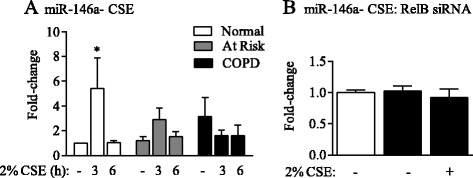
Regulation of miR-146a by CSE is independent of RelB. **(A)** miR-146a- CSE: 2% CSE significantly increased miR-146a expression only in Normal lung fibroblasts at 3 hours (fold-induction: 5.44 ± 2.4; * p < 0.05). Results are expressed as the mean ± SEM of normalized miR-146a levels; n = 4 independent experiments utilizing fibroblasts from 4 different subjects of each phenotype; post-hoc analysis was performed by Fisher’s LSD. **(B)** miR-146a-CSE RelB siRNA: There was no significant difference in basal or CSE-exposed (3 hrs) miR-146a levels in siRelB cells (*black bars*) compared to siCtrl cells (*open bars*). Results are expressed as the mean ± SEM of normalized miR-146a levels from 2–4 independent experiments.

We recently published that RelB promotes the induction of miR-146a in response to cigarette smoke in murine lung fibroblasts as a mechanism through which RelB limits COX-2 protein levels [31]. COPD lung fibroblasts have reduced miR-146a induction in response to pro-inflammatory cytokines, a finding that correlated with increased COX-2 expression [36]. These findings led us to speculate that RelB control over smoke-induced COX-2 expression occurs via miR-146a. While there was significantly more basal miR-146a in COPD lung tissue compared to Normal (Figure 7A), there was no difference in basal levels of miR-146a between the three fibroblast groups (Figure 7B). In Normal fibroblasts, but not At Risk or COPD cells, there was a significant increase in miR-146a after exposure to 2% CSE for 3 hours (Figure 8A), the peak expression time for miR-146a in response to cigarette smoke [31]. Knock-down of RelB had no effect on miR-146a (Figure 8B) suggesting regulation of miR-146a expression by cigarette smoke is independent of RelB in human lung fibroblasts.

### Correlation between systemic RelB, Cox-2 and miR-146a expression and clinical features of COPD in the CanCOLD cohort

**Figure 9:**
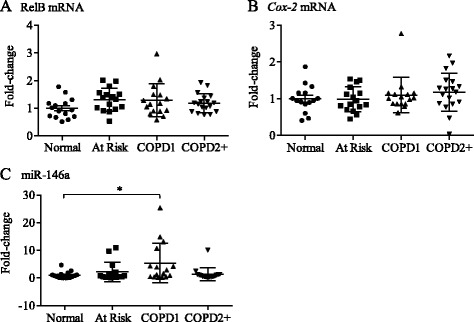
Systemic expression of *Cox-2*, RelB and miR-146a levels in COPD. Peripheral blood was obtained from subjects recruited as part of the CanCOLD project and total RNA, including miRNA, was isolated and the expression of *Cox-2* and RelB mRNA as well as miR-146a evaluated by qRT-PCR. A total of 16 subjects from each category were analyzed from the CanCOLD cohort; each symbol represents a different individual. **(A)** RelB mRNA- there was no significant difference in the relative expression of RelB mRNA between the subject categories. **(B)** *Cox-2* mRNA: there was no significant difference in the relative expression of RelB mRNA between the subject categories. **(C)** miR-146a: there was a significant increase in relative miR-146a expression in COPD Gold 1 compared to Normal subjects.

**Figure 10:**
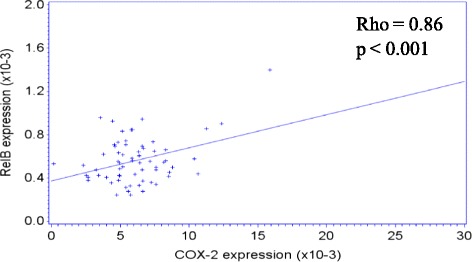
Correlation between systemic RelB and *Cox-2* mRNA expression in COPD. A total of 16 subjects from each category were analyzed from the CanCOLD cohort. There was a significant positive correlation between systemic RelB and *Cox-2* mRNA expression.

We recently published that RelB expression correlates with clinical features of COPD exacerbations [55]. To next establish if there was correlation between systemic expression of RelB, *Cox-2* and miR-146a in COPD, we utilized the CanCOLD cohort. Of these subjects, there was no significant difference in terms of age and gender between the 4 study groups (Table 2). RelB and *Cox-2* expression were not significantly different between the subject groups whereas the relative expression of miR-146a approached statistical significance (p = 0.064) (Table 2). Neither RelB nor *Cox-2* mRNA expression correlated with the clinical variables evaluated in this population, including lung function (Table 3). However, miR-146a significantly correlated with FEV_1_% predicted (p = 0.026) and trended towards significance for FEV_1_/FVC (p = 0.065) (Table 3). There was a significant increase in systemic miR-146a in COPD I (GOLD 1) compared to Normal (Figure 9). There was also a significant positive correlation between RelB and *Cox-2* expression (Table 3 and Figure 10). Collectively these data highlight a differential role for systemic versus pulmonary RelB expression in COPD and suggest the importance of lung structural cell RelB expression in regulating inflammation caused by smoke exposure.

**Table 3:** **Pearson correlation coefficients between biomarkers and clinical variables**

**Variable 1**	**Variable 2**	**Correlation coefficient**	**95% CI**	**P-value**
RelB expression	Cox-2 expression	0.86	0.78 - 0.91	<0.001**
RelB expression	146a	−0.08	−0.32 - 0.16	0.509
RelB expression	FEV_1_ L	−0.04	−0.28 - 0.21	0.768
RelB expression	FEV_1_% predicted	0.05	−0.19 - 0.29	0.671
RelB expression	FEV_1_/FVC	0.10	−0.15 - 0.34	0.418
Cox-2 expression	146a	−0.12	−0.36 - 0.12	0.323
Cox-2 expression	FEV_1_ L	0.01	−0.24 - 0.25	0.931
Cox-2 expression	FEV_1_% predicted	0.09	−0.16 - 0.33	0.475
Cox-2 expression	FEV_1_/FVC	0.10	−0.15 - 0.34	0.428
146a	FEV_1_ L	−0.20	−0.42 - 0.05	0.114
146a	FEV_1_% predicted	−0.28	−0.49 -0.04	0.026*
146a	FEV_1_/FVC	−0.23	−0.45 - 0.01	0.065

**indicates significant correlation between RelB and Cox-2 expression.

## Discussion

COPD is an obstructive lung disease that is increasing in prevalence worldwide, affecting an estimated 200 million people [56]. While the etiology of COPD is strongly linked to smoke exposure, the underlying pathogenic mechanisms by which smoke causes chronic, aberrant pulmonary inflammation remains poorly defined. Numerous signal transduction pathways, including the classic (p65/p50) NF-κB pathway, contribute to cigarette smoke-induced inflammation. The pro-inflammatory activities of the classic NF-κB pathway are counterbalanced by another REL protein called RelB, initially identified as I-Rel for Inhibitory Rel because of its ability to reduce the transcriptional activities of NF-κB [47]. We have published that over-expression of RelB diminishes the induction of inflammatory mediators, including COX-2 as well as consequent lung neutrophilia caused by cigarette smoke [29,30]. These finding highlight RelB as a potentially important anti-inflammatory REL protein that protects against the deleterious effects of cigarette smoke, raising the possibility that low/absent RelB levels in the lung may predispose some individuals who smoke to aberrant inflammation and the eventual development of COPD. To our knowledge, we are the first to demonstrate that lung fibroblasts from At Risk (smokers with no airflow obstruction) and COPD subjects have significantly less RelB mRNA and protein expression compared to fibroblasts from individuals who are non-smokers (Normal).

One of the more intriguing findings from our study is that while there was a significant decrease in RelB mRNA in lung fibroblasts from At Risk and COPD subjects (Figure 2), there was no significant change in systemic RelB mRNA levels based on smoking status or airflow limitation (Table 2). This latter observation is consistent with our recent publication in which we demonstrated that systemic RelB expression was associated with health outcomes during acute exacerbations in COPD but was not associated with clinical features during stable-state [55]. While this difference between pulmonary and systemic RelB expression may be reflective of the fact that the lung fibroblasts were derived from different subjects compared to those from which systemic RelB was measured, we postulate that this disparity is reflective of fundamental differences in RelB expression, activation and function between structural and immune cells. Whereas RelB is highly expressed and constitutively active in lymphoid cells [57], there is minimal RelB activity in quiescent structural cells such as endothelial cells and fibroblasts [24,58]. In response to certain inflammatory triggers however (*e.g.* IL-1β, lipopolysaccharide [LPS], CD40L), RelB expression and/or activity is increased which serves to dampen the expression of inflammation-associated proteins such as chemokines and adhesion molecules [24,29,59]. This is not the case with immune cells, where RelB does not control the production of chemokines from LPS-stimulated macrophages [59]. Moreover the anti-inflammatory abilities of RelB in protecting against inflammation are largely attributed to non-hematopoietic cells, particularly fibroblasts. Evidence in support of this includes: (1) the transfer of normal bone marrow into irradiated RelB-deficient mice failed to alleviated the inflammatory syndrome and (2) injection of LPS-stimulated RelB^−/−^ fibroblasts potently induced inflammation *in vivo* [59]. Thus, it may be that lung fibroblasts with low RelB expression due to chronic smoke exposure (rather than immune cells) are active participants in the abnormal inflammation associated with cigarette smoke exposure, capable of inciting and perpetuating the pulmonary inflammatory response via the production of inflammatory mediators such as COX-2.

Our observation that lung fibroblasts from individuals who smoke (At Risk) and those with airflow limitation (COPD) have reduced RelB mRNA and protein expression (Figure 2) suggests that cigarette smoke directly reduces RelB levels, a notion further supported by our *in vitro* experimental data showing CSE exposure decreases RelB protein (Figure 3). Our data also support that the mechanism by which smoke decreases RelB protein is due to degradation by the 26S proteasome (Figure 3D). Consistent with this notion, others have demonstrated that RelB is proteolytically degraded in T cells in a ligand-specific manner [50,60], including the appearance of the lower MW band of ≈ 55 kDa (Figure 2A) [49] and that RelB degradation also occurs in pulmonary cells exposed to hypercapnia [25]. Another unresolved question is why there was a significant decrease in RelB mRNA in smoker-derived (At Risk) lung fibroblasts (Figure 2) whereas exposure to CSE *in vitro* did not significantly decrease RelB mRNA levels in any of the subject groups (Figure 3). It is possible that chronic long-term exposure to cigarette smoke, characteristic of individuals in the At Risk and COPD categories, is necessary to alter RelB mRNA expression, such that a single exposure to cigarette smoke over a 24 hour time-period, our standard *in vitro* exposure protocol, is insufficient to alter RelB at the mRNA level.

It is somewhat paradoxical that there was a significant cigarette smoke-induction of *Cox-2* mRNA only in COPD cells (and not At Risk), despite the fact that both have similar low RelB expression levels (Figures 2 and 4). At first glance this might indicate that RelB ultimately does not contribute to the regulation of smoke-induced COX-2. However our data utilizing siRNA to knock-down RelB in Normal cells, where there is potentiation of CSE-induced *Cox-2* mRNA expression (Figure 5), expands data obtained from our *in vitro* and *in vivo* models of smoke exposure [30] to show that RelB contributes to the suppression of cigarette smoke-induced COX-2 in human lung cells. One explanation for these data is that RelB is in fact critical in attenuating COX-2 in naive cells that initially encounter respiratory toxicants such as cigarette smoke, but that RelB alone is insufficient in counter-balancing the deleterious effects associated with chronic, long-term smoke exposure in COPD subjects. It may also be that post-translational modifications of RelB protein occur in COPD, accounting for the differential response between At Risk and COPD-derived cells, both of which have reduced RelB protein levels but divergent transcriptional changes in *Cox-2* mRNA. Protein ubiquitination, which is implicated in COPD pathogenesis [61], augments the transactivation potential of RelB to promote NF-κB-dependent transcription [62]. Thus if such modifications account for the difference in response between At Risk and COPD cells, then alteration of RelB levels (via siRNA) in At Risk fibroblasts would be ineffectual in altering smoke-induced *Cox-2* expression. There is also no difference in the expression or nuclear localization of p65 between the three groups in response to CSE (Figure 6), making it unlikely that RelB suppression of *Cox-2* is via alteration in the canonical NF-κB pathway. It remains possible that co-activators such as p300, necessary for transcriptional induction of *Cox-2* by NF-κB [63], are repressed by RelB or altered in COPD, thereby accounting for differences in *Cox-2* between At Risk and COPD fibroblasts or the initiation of *Cox-2* transcription after RelB knock-down. Moreover, cigarette smoke exposure can cause epigenetic changes in the lung, leading to significant increases in inflammatory proteins associated with COPD pathogenesis [64,65]. Thus it could also be that, in addition to low RelB levels in COPD, there are further epigenetic changes in the lung or additional protein modifications to the RelB protein not identified in this study that render it unable to exert negative control over repeated/chronic exposures. Such epigenetic alterations could also be why there is reduced RelB mRNA in lung fibroblasts from chronic smokers but not after a single 24-hour exposure. Finally, another potential explanation is that a protein partner of the RelB pathway essential for its full inhibitory activities (*e.g.* p100/p52 or the aryl hydrocarbon receptor [AhR]) [29] may also be inherently absent or defective in COPD subjects, and thus not allow for the full anti-inflammatory abilities of RelB. These and other possibilities are currently being explored.

Recently, a reciprocal relationship between RelB and miR-146a in immunity and inflammation has emerged including the induction of miR-146a by IL-1β [66] and CSE [31]. McMillan and colleagues demonstrated that in adult lung fibroblasts, downregulation of RelB via siRNA decreases the magnitude of IL-1β-induced miR-146a expression [66]. Sato *et al.* demonstrated that COPD fibroblasts produce less cytokine-stimulated miR-146a compared to fibroblasts from smokers (At Risk) [36]. In our study, both At Risk and COPD fibroblasts also failed to significantly increase miR-146a in response to CSE, and effect that was independent of RelB expression (Figure 8), suggesting that RelB does not contribute to CSE-induction of miR-146a in human lung fibroblasts. This differs from our recently published data utilizing RelB^−/−^ mouse lung fibroblasts, which has significantly less miR-146a compared to RelB-expressing cells [31]. While this may reflect species-specific differences in basal miR-146a regulation, it is equally likely that even low detectable levels of RelB expression in At Risk and COPD fibroblasts are sufficient to promote basal miR-146a expression. Of the potential systemic markers examined in this study, only miR-146a was associated with health outcomes (Table 3) and increased in COPD (Figures 7 and 9). Our findings that basal miR-146a was higher in both COPD lung tissue as well as systemically in the blood, but not in lung fibroblasts, suggests that cells of hematopoietic origin- and not lung structural cells- contribute to this heightened expression. It is also intriguing that miR-146a was significantly increased in COPD Gold 1 but not with more severe disease (Gold 2+). Whether the increased miR-146a in COPD 1 is a compensatory mechanism or predictive of those individuals who will go on to develop more severe COPD is not known. Further longitudinal assessment of miR-146a utilizing the CanCOLD population may reveal the novelty of miR-146a as a biomarker of COPD progression.

We recognize that there are several limitations with our study, including the cross-sectional nature of the data obtained from the fibroblasts derived from the lung surgical specimens. This ultimately makes us unable to determine if the individuals who are smokers with low RelB will ultimately develop COPD. We also cannot exclude the possibility that medications (*e.g.* inhaled corticosteroids) taken by the subjects in our study had an impact on the relative expression levels of RelB, as corticosteroids can dampen *Cox-2* gene transcription via NF-κB [67]. Finally, another perceived limitation is the reliance on mRNA levels to correlate with clinical parameters, as quantification of blood RelB protein expression remains to be determined. Despite these limitations, our data support that RelB suppresses COX-2 expression upon exposure to cigarette smoke. When considered with our previous work [31], our data implies that RelB and miR-146a may work cooperatively to suppress COX-2 expression in response to environmental toxicants.

## Conclusions

To the best of our knowledge, we are the first to report on the expression of RelB in primary lung fibroblasts derived from COPD, and show that cigarette smoke contributes to a reduction in RelB expression in lung structural cells. Our data further demonstrate the importance of RelB expression in attenuating COX-2 protein in response to *in vitro* exposure to cigarette smoke extract. Whether low RelB levels predispose to the development of COPD in susceptible individuals is not known but further molecular investigation into the alternative NF-κB pathway will enhance our understanding of RelB in COPD and may contribute to the development of novel, lung-targeted anti-inflammatory treatments for smoke-related lung disorders.

## Abbreviations

CanCOLD: Canadian chronic obstructive lung disease
COPD: Chronic obstructive pulmonary disease
COX-2: Cyclooxygenase-2
miRNA: MicroRNA
NF-κB: Nuclear factor-κB
